# Supramolecular Controlled Cargo Release *via* Near Infrared Tunable Cucurbit[7]uril-Gold Nanostars

**DOI:** 10.1038/srep22239

**Published:** 2016-02-26

**Authors:** Yanwei Han, Xiran Yang, Yingzhu Liu, Qiushuang Ai, Simin Liu, Chunyan Sun, Feng Liang

**Affiliations:** 1 The State Key Laboratory of Refractories and Metallurgy, Wuhan University of Science and Technology, Wuhan 430081, China; 2 College of Chemical Engineering and Technology, Wuhan University of Science and Technology, Wuhan 430081, China; 3 Key Laboratory of Analytical Chemistry for Biology and Medicine (Ministry of Education), Wuhan University, Wuhan 430072, China; 4 Institute of Hematology, Union Hospital, Tongji Medical College, Huazhong University of Science and Technology, Wuhan 430022, China

## Abstract

The near infrared (NIR) absorption and average particle size of gold nanostars (GNSs) can be precisely controlled by varying the molar ratios of cucurbit[7]urils (CB[7]) and GNSs in aqueous solution. GNSs modified with CB[7] achieved high cargo loading with thermally activated release upon the NIR laser irradiation.

Gold nanoparticles (GNPs) have been intensely investigated over the past decades because of their facile synthesis, biocompatibility, chemical stability and unique optical properties^1^,^2^. A variety of gold nanoparticles, such as gold nanorods, nanoshells, nanocages, nanotripods, nanohexapods, nanobellflowers, nanoflowers, and nanostars have been proved to exhibit plasmon resonance in the near-infrared (NIR) region (700–1100 nm)^3^,^4^,^5^,^6^,^7^,^8^ which can easily penetrate into living organisms with minimally invasive effects, and produce hyperthermia upon the NIR laser irradiation with high efficiency^9^,^10^. Among these geometries, nanostars (GNSs) have received a great deal of interest because multiple sharp branches could be more effective in photothermal conversion and cargo loading efficiency relative to those with smooth surfaces owing to their high surface-to-volume ratios^11^,^12^,^13^.

Cucurbit[*n*]urils (CB[*n*], Fig. 1) are a group of cyclic, pumpkin-shaped molecules composed of n glycoluril units (*n* = 5–8, 10, 14) bridged by methylene groups, that form stable host-guest inclusion complexes with a wide variety of guest molecules in aqueous media^14^,^15^,^16^. The CB[*n*] family offers remarkably higher selectivity over conventional macrocyclic receptors towards the shape and charge of many types of guests in aqueous media, driving by a combination of ion dipole interactions, hydrogen bonds, and hydrophobic effect^17^,^18^,^19^. Because of their inherent modularity and reversibility, supramolecular CB[*n*] systems can be further engineered to assemble and disassemble spontaneously in response to a range of triggers^20^,^21^,^22^,^23^.

Since Li’s group reported that native CB[*n*]s could strongly bind to the flat gold surfaces by carbonyl-fringed portals^24^, Scherman’s group has significantly developed synthetic methodology for gold nanoparticles capped with CB[*n*] (where *n* = 5–8) in aqueous media^25^,^26^. The gold aggregates could be fabricated with uniform nanogaps through the assembly of the macrocycle CB[*n*] with gold colloids^27^,^28^,^29^. Both the gap distance and the length of the assembled chain control large optical field enhancements and well defined chain-like plasmon modes that extend across the visible and infrared spectrum^30^,^31^. Recently, Scherman’s group demonstrated the real-time Surface-enhanced Raman scattering (SERS) monitoring of a prototypical stilbene photoreaction in a CB[*n*]-GNPs nanoreactor^32^. Brunsveld’s group achieved supramolecular control of cell adhesion *via* CB[*n*] based host-guest systems on gold arrays^33^.

Although cucurbit[*n*]urils are capable of reproducibly controlling the desired distance between gold nanospheres and nanorods by acting as rigid spacers^24^,^25^,^26^,^27^,^28^,^29^,^30^,^31^,^32^, most of reported works focused on CB[*n*] aligned gold aggregate and few attention was paid to highly stable CB[*n*]-GNPs systems in solution without needing additional organic ligands or metallic cations^34^,^35^. It also has been approved that CB[7] could be used in the creation of drug delivery vectors^36^, regulating exocytosis of gold nanoparticles and activation of therapeutic gold nanoparticles inside living cells^37^,^38^. As a result, the present work was motivated by developing a remarkable CB[7]-GNPs cargo release system with both autonomous recognition and external control because our long-term goal is to construct multifunctional drug delivery systems by using macrocyclic receptors and nanoparticles^39^,^40^,^41^.

## Results and Discussion

### Synthesis and characterization of GNSs capped with CB[7]

Firstly, we prepared monodisperse gold nanostars according to a surfactant-free wet-chemistry method^42^ in order to avoid the potential toxicity of surfactants and the difficulty of replacing the surfactants, such as poly(N-vinylpyrrolidone) (PVP) and cetyltrimethylammonium bromide (CTAB)^43^,^44^. The synthesis simply and quickly resulted in gold nanostars of around 100 nm with narrow size distribution (Fig. S1). Because lack of optical experimental measurements of gold nanostars^45^,^46^, the concentration of GNSs was roughly calculated based on the measurement of transmission electron microscope (TEM) and inductively coupled plasma optical emission spectroscopy (ICP-OES)^47^,^48^.

**Figure 2:**
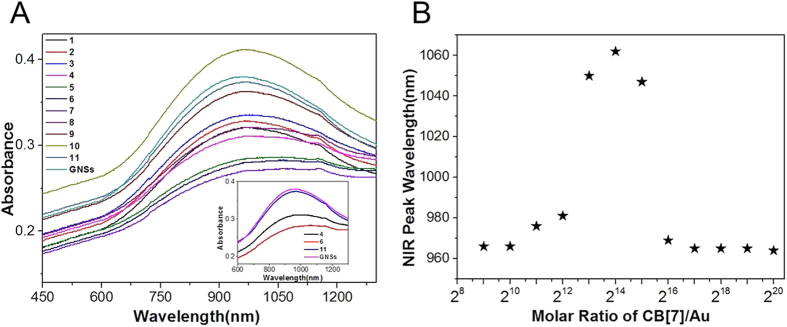
(**A**) UV-vis-NIR spectrum of the CB[7]-GNSs with CB[7]/Au ratio = (1) 2^9^, (2) 2^10^, (3) 2^11^, (4) 2^12^, (5) 2^13^, (6) 2^14^, (7) 2^15^, (8) 2^16^, (9) 2^17^, (10) 2^18^ and (11) 2^19^ by addition of CB[7]; (**B**) NIR peak wavelength as functions of CB[7]/Au ratio.

The modification of GNSs by CB[7] was monitored by UV-vis-NIR absorption spectra. In the UV-vis-NIR spectra of the sample solutions shown in Fig. 2, GNSs showed a distinct absorption peak at 964 nm. Consistent with the previous reports^25^, the distinct absorption peak could be red-shifted to 1062 nm (at CB[7]/GNSs ratio = 2^14^: 1, sample 6) after the addition of CB[7], implying the persistence of the aggregates induced by CB[7]. Upon further increase in the ratio of CB[7] to GNSs (CB[7]/ GNSs up to 2^19^: 1, sample 11), the distinct absorption peak shifted back to that of GNSs (Table S1). This suggested that the GNPs decreased in size with an increasing amount of CB[7] in solution, which was in agreement with the following TEM and Laser Particle Size results.

**Figure 3:**
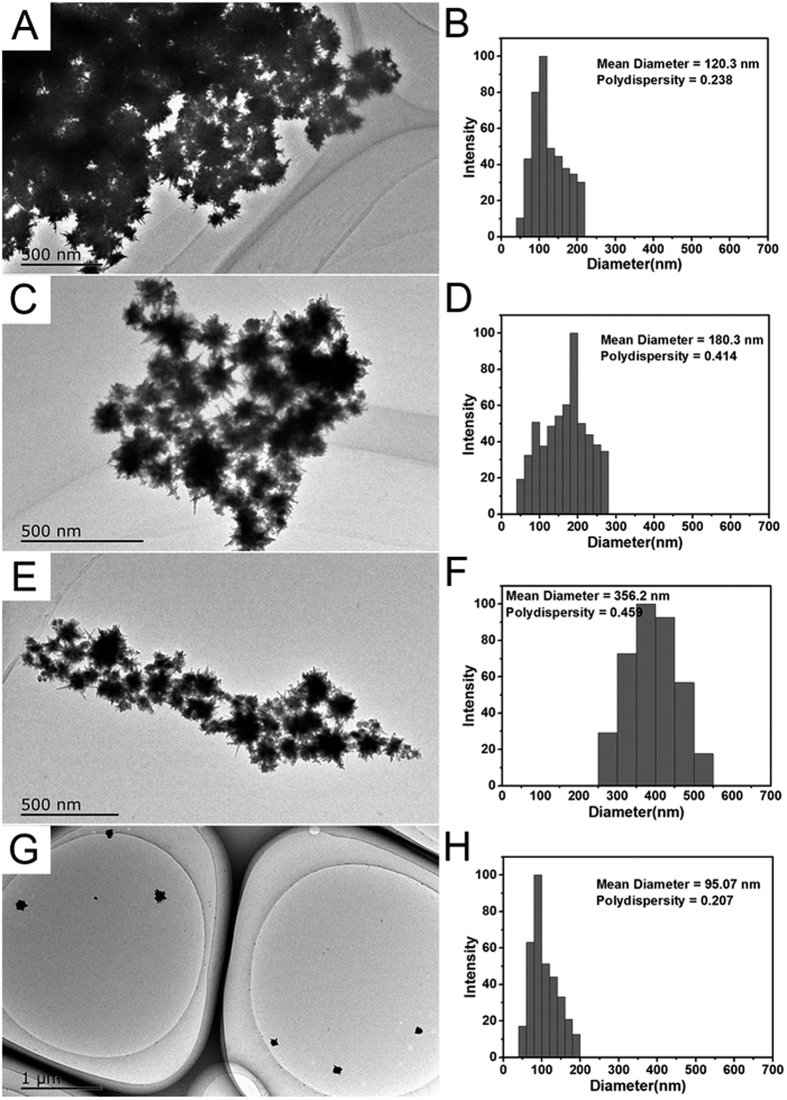
TEM images of GNSs (**A**) and CB[7]-GNSs with CB[7]/Au ratio = (**C**) 2^12^, sample 4; (**E**) 2^14^, sample 6; (**G**) 2^19^, sample 11 and hydrodynamic diameter of GNSs (**B**) and CB[7]-GNSs with CB[7]/Au ratio = (**D**) 2^12^, sample 4; (**F**) 2^14^, sample 6; (**H**) 2^19^, sample 11 measured by laser particle size analyzer.

The morphology and size of the obtained GNS and CB[7]-GNSs were examined by TEM. Figure 3 shows the representative images of the nanoparticles surface-functionalized with CB[7], which have nearly uniform sizes. Without the addition of CB[7], the GNSs was easily aggregated together. With the increasing of CB[7], GNSs was gradually dispersed from cluster to single nanoparticle.

Laser particle size analysis was employed to probe the size of the dynamic, self-assembled structures in solution quantitatively. The hydrodynamic diameters of GNS and CB[7]-GNSs (Figs 3 and S2) were 120.3 nm (polydispersity index: 0.238, GNSs), 180.3 nm (polydispersity index: 0.414, CB[7]/GNSs ratio = 2^12^: 1, sample 4), 356.2 nm (polydispersity index: 0.459, CB[7]/GNSs ratio = 2^14^: 1, sample 6), and 95.1 nm (polydispersity index: 0.207, CB[7]/ GNSs ratio = 2^19^: 1, sample 11) respectively. It was clear that the dispersion ability depended significantly on the CB[7] density on the GNSs surface. Thus, CB on the gold surface served as a stabilizer to impart the nanoparticles with exceptional dispersibility and stability.

Zeta potential of nanoparticles were recorded, as shown in Fig. S3. Zeta potential values of CB[7]-GNSs with different molar ratio are −10.5, −10.1, +7.1, and +12.1 mV at pH 5.0, respectively, gradually turning to positive charges with increased CB[7]. These data are consistent with the fact that CB[7]-GNPs were stabilized by positive surface charges. Owing to the electrostatic repulsion effect, the representative CB[7]-GNPs showed complete dispersion stability, avoiding coagulation in aqueous solution^34^. The excellent dispersion stability of the obtained nanoparticles was also confirmed both in phosphate buffer solution and cell culture medium. No visible sedimentation was observed after standing for more than 3 days at room temperature (Fig. S4).

It has been calculated that the surface of a nanosphere with a diameter of 8 nm could accommodate a maximum of 25 molecules of CB[7]^49^, thus the gold nanosphere with a diameter of 100 nm would be able to accommodate a maximum of around 49,000 molecules of CB[7]. As for sample 11 in our case, an average number of around 524,000 (2^19^) CB[7] molecules per nanoparticle can be calculated. This result is in reasonable agreement with the prediction that nanostar with branches could load much more molecules of CB[7] than nanosphere. GNSs capped with CB[7] were also verified by Fourier transform infrared (FT-IR) spectroscopy. As shown in Fig. S5, a typical absorption peak at 1726 cm^−1^ was assigned to C=O stretching at the CB[7] portals. In contrast, there are two C=O stretching bands at 1732 (small) and 1643 cm^−1^ (big) for CB[7]-GNSs samples. It was also observed that an absorption peak at 1478 cm^−1^ due to C-N stretching for free CB[7] red-shifted to 1396 cm^−1^ in CB[7]-GNSs samples. These shifts are very similar to that of magnetic Fe nanoparticles capped with CB[n]^49^,^50^. It was also noticed that in our case the red-shift was more significant (82 cm^−1^) than that reported by Scherman’s group (15 cm^−1^)^25^.

### Photothermal conversion of nanoparticles

**Figure 4:**
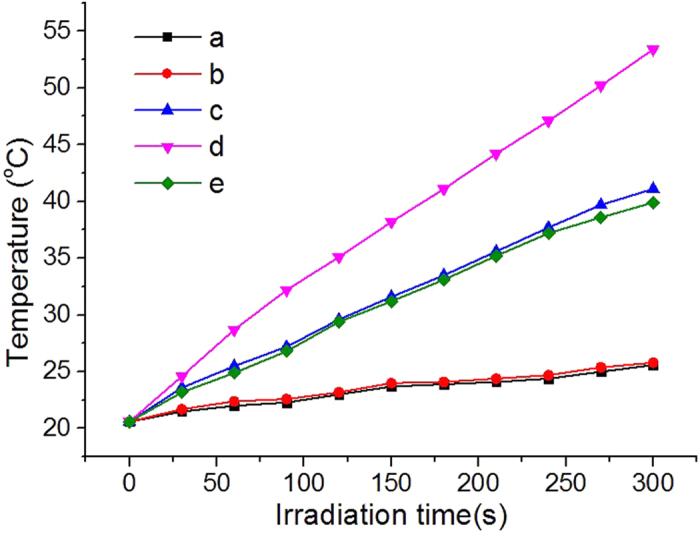
Plots of temperatures as a function of irradiation time for solutions (**a**) without CB[7] and GNSs, (**b**) with CB[7], (**c**) with GNSs, (**d**) with sample 6, and (**e**) with sample 11.

We then compared the photothermal conversion efficiencies of nanoparticles by measuring the temperature rise for their aqueous solutions upon laser irradiation. Briefly, the GNSs and CB[7]-GNSs solutions (0.7 nmol/L) were each added into different wells of the 12-well plate, and the radiation was delivered using a diode laser centered at 808 nm from the top at a density of 1 W/cm^2^. The results demonstrated that the aqueous solutions containing of the nanostructures generated significant temperature increases upon excitation. As shown in Fig. 4, the solution of sample 6 (with the largest particle size and the longest distinct absorption peak wavelength) showed a rapid increase in temperature during 5 min and eventually reached a total temperature increase of 32.8 °C. The rate of temperature rise and the final temperature were proportional to the particle size and NIR peak wavelength; typically a faster and bigger increase. In the absence of any Au nanoparticles, the solution increased in temperature by only 5.2 °C and 5 °C with and without CB[7] (at the concentration of 0.7 × 2^19 ^nmol/L, same with that in sample 11) after 5 min of constant irradiation under similar conditions.

### Cargo transportation of GNSs-CB[7]

**Figure 5:**
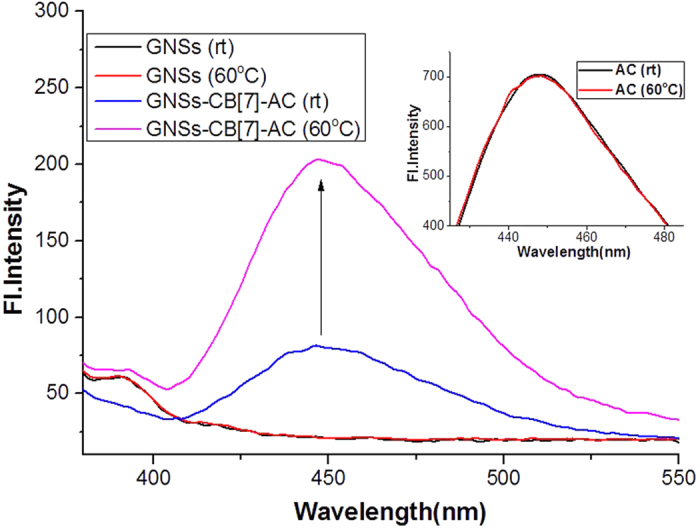
Fluorescence spectra of GNSs, 6-AC and GNSs-CB[7]-AC at different temperatures in aqueous solution (λ_ex_ = 347 nm). Fluorescence spectra were measured on a PerkinElmer LS-55 machine.

6-Aminocoumarin (6-AC) is a strong fluorescent compound, which was used as cargo in our loading-releasing system since the change of its concentration can be monitored by the fluorescence spectrometer at low concentration. First we tested the binding between 6-AC and CB[7]. As we expected, 6-AC could form stable 1:1 host-guest complex with CB[7] (*K*_*a*_ = 8.96 × 10^3^ M^−1^ at 30 °C) (Fig. S6). The binding strength of 6-AC and CB[7] dramatically decreased with the increasing of temperature (Table S2), which could make the idea of releasing the cargo by photothermal conversion possible. Then we verified that 6-AC can be loaded in GNSs-CB[7] system. TGA (thermogravimetric analysis) is very helpful in proving the loading of 6-AC. As shown in Fig. S7, an additional weight loss that corresponds to 6-AC is observed, which clearly testify to the successful encapsulation of 6-AC into the cavities of CB[7]. Further we did variable temperature experiments to check whether 6-AC as the cargo could be released from this system. As we can see in Fig. 5, due to the loading on the particle of GNSs-CB[7], fluorescence intensity of 6-AC was quite low. And fluorescence intensity of GNSs or 6-AC themselves didn’t change at all with the increasing of temperature. In the case of GNSs-CB[7]-AC system, increased fluorescence signal could be discerned with the increased temperature from rt to 60 °C, which implied that 6-AC was truly released from this system.

## Conclusion

In conclusion, it has been demonstrated that NIR absorption and average particle size of gold nanostars could be precisely controlled by varying the molar ratios of cucurbit[7]urils and GNSs in aqueous solution. CB[7]-GNSs exhibit strong absorption in the NIR region, relatively high photothermal conversion efficiency and high cargo loading with controllable release. CB[7]-GNSs, with the unique properties of both CB[n] and gold nanoparticles, therefore is a promising photothermal conversion agent (PTCA) and cargo transporter^51^,^52^,^53^, which could be further developed and applied in biomedicine and biotechnology. Related research project is in progress in our groups.

## Additional Information

**How to cite this article**: Han, Y. *et al*. Supramolecular Controlled Cargo Release *via* Near Infrared Tunable Cucurbit[7]uril-Gold Nanostars. *Sci. Rep*. **6**, 22239; doi: 10.1038/srep22239 (2016).

## Supplementary Material

Supplementary Information

## Figures and Tables

**Figure 1:**
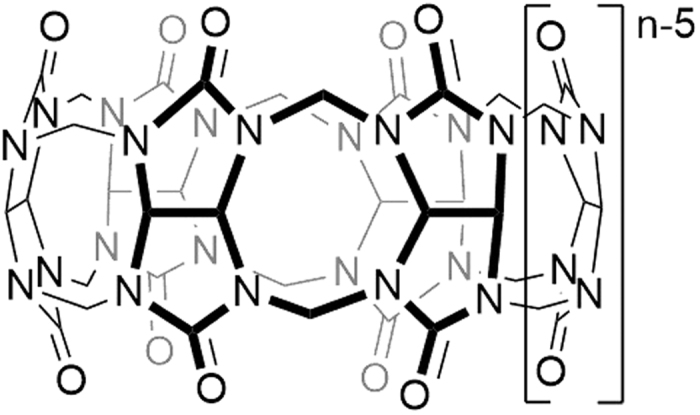
Schematic representation of Cucurbit[*n*]urils (CB[*n*]).
