# Supplementary material for: Supramolecular Controlled Cargo Release via Near Infrared Tunable Cucurbit[7]uril-Gold Nanostars
Source: Sci Rep. 2016 Feb 26;6:22239. doi: 10.1038/srep22239 (PMC4768098; doi:10.1038/srep22239)
Supplement: Supplementary Information [file srep22239-s1.pdf]

Supporting Information

for

Supramolecular Controlled Cargo Release *via* Near Infrared Tunable Cucurbit[7]uril-Gold Nanostars

Yanwei Han[b, †], Xiran Yang[b, †], Yingzhu Liu[b], Qiushuang Ai[b], Simin Liu[*a, b], Chunyan Sun[d] and Feng Liang[* a, b, c]

[a.] *The State Key Laboratory of Refractories and Metallurgy, Wuhan University of Science and Technology, Wuhan 430081, China.*
[b.] *College of Chemical Engineering and Technology, Wuhan University of Science and Technology, Wuhan 430081, China.*
[c.] *Key Laboratory of Analytical Chemistry for Biology and Medicine (Ministry of Education), Wuhan University, Wuhan 430072, China.*
[d.] *Institute of Hematology, Union Hospital, Tongji Medical College, Huazhong University of Science and Technology, Wuhan 430022, China*

*** To whom correspondence should be addressed,**
E-mail: chemliusm1976@yahoo.com (S. Liu) or feng_liang@whu.edu.cn (F. Liang)

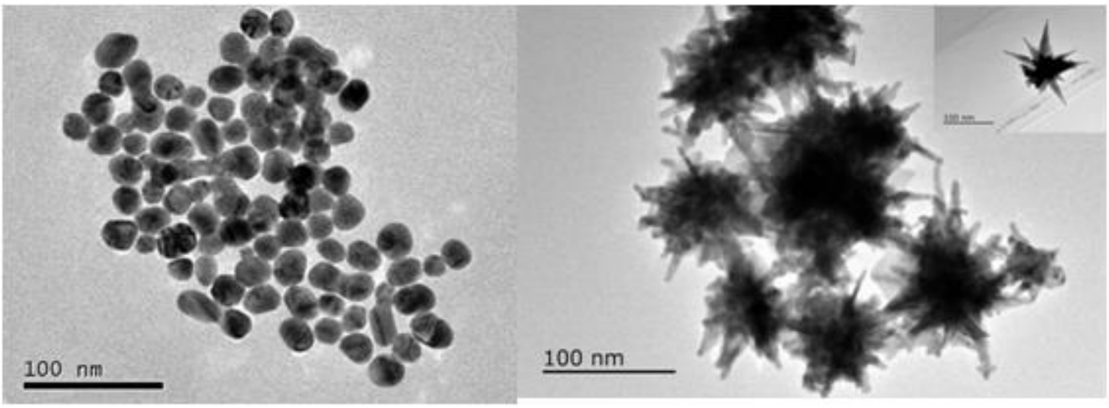

Fig. S1 Typical TEM images of synthesized gold nanospheres (0.7 nM in particle concentration or 394.9 μg/mL of Au atoms) and gold nanostars in this work (0.07 nM in particle concentration or 37.50 μg/mL of Au atoms and 2.45 μg/mL of Ag atoms). The elemental analyses were performed using inductively coupled plasma optical emission spectroscopy (ICP-OES, IRIS Advantage Duo ER/S spectrometer, Thermo Jarrell Ash, USA). Regular NP samples were suspended in freshly prepared aqua regia (trace metal grade 70% nitric acid HNO_3:36% hydrochloric acid HCl, 1:3/v:v) and heated until completely dissolved, and then diluted with double-distilled water.

Table S1 Optical Properties of GNSs and CB[7]-GNSs[§]

Samples	$C_{GNSss}:C_{CB[7]}$	NIR peak wavelength/nm
**1**	$1:2^{9}$	966
**2**	$1:2^{10}$	966
**3**	$1:2^{11}$	976
**4**	$1:2^{12}$	981
**5**	$1:2^{13}$	1050
**6**	$1:2^{14}$	1062
**7**	$1:2^{15}$	1047
**8**	$1:2^{16}$	969
**9**	$1:2^{17}$	965
**10**	$1:2^{18}$	965
**11**	$1:2^{19}$	965
**GNSs**	/	964

[§] The fresh made GNSs solution was added CB[7] (1 mM) solution with pipette (Eppendorf) to produce desired CB[7]-GNSs. The absorption spectra were acquired with a UV-3600 UV-vis-NIR Spectroscopy (Shimadzu, Japan).

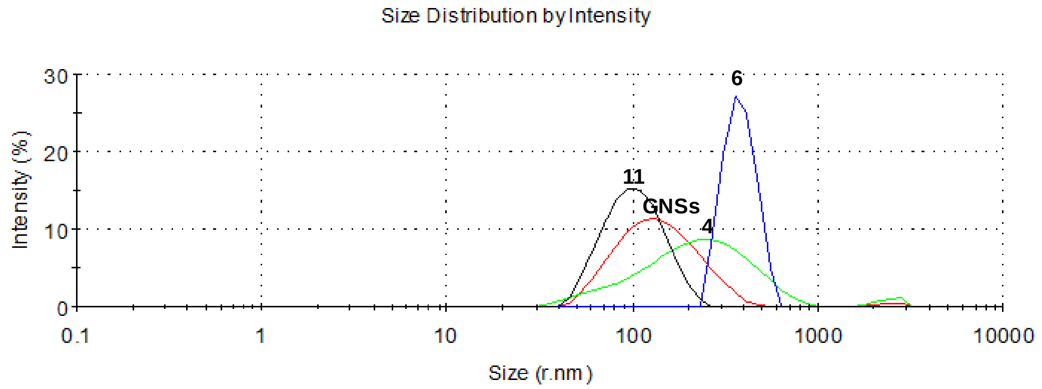

Fig. S2 Hydrodynamic diameter of GNSs (red) and CB[7]-GNSs with CB[7]/Au ratio = 2^{12}, sample 4 (green); 2^{14}, sample 6 (blue) and 2^{19}, sample 11 (black) measured by Zetasizer Nano ZS ZEN3600 (Malvern, UK). Polydispersity values were utilized to evaluate the distribution of the nanoparticles population.

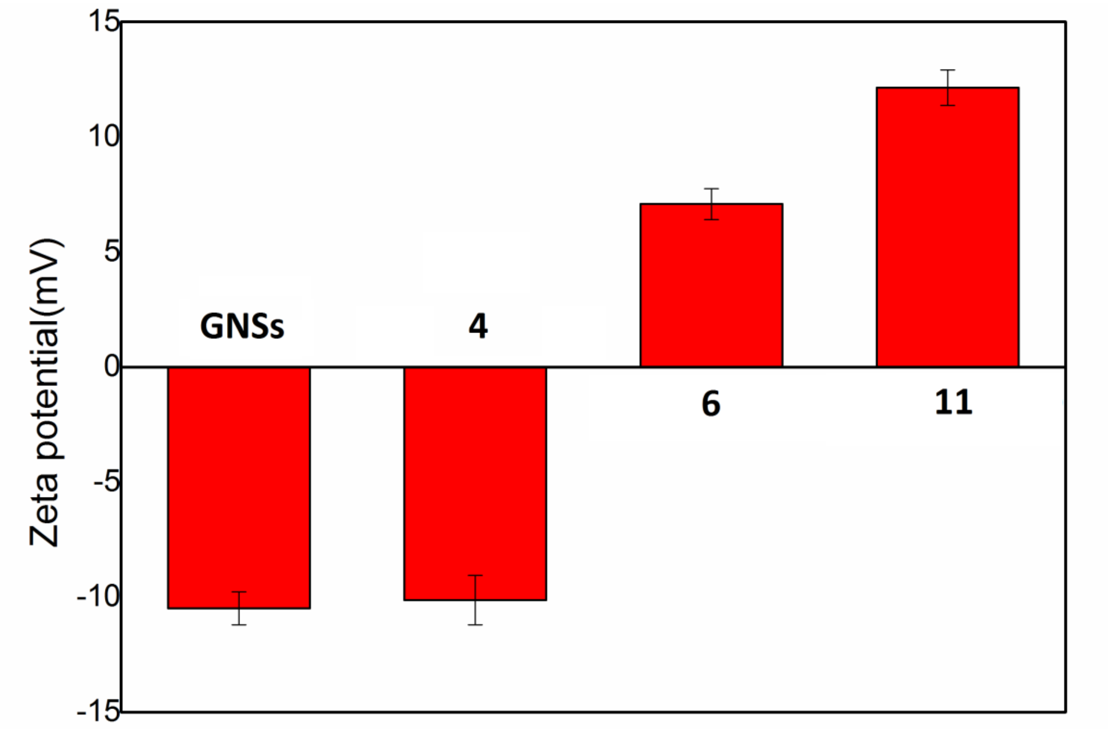

Fig. S3 Zeta potential of GNSs and CB[7]-GNSs with CB[7]/Au ratio = 2^{12}, sample 4; 2^{14}, sample 6 and 2^{19}, sample 11 measured by Zetasizer Nano ZS ZEN3600 (Malvern, UK) following manufacturer instructions. Each value reported is the average of three consecutive measurements.

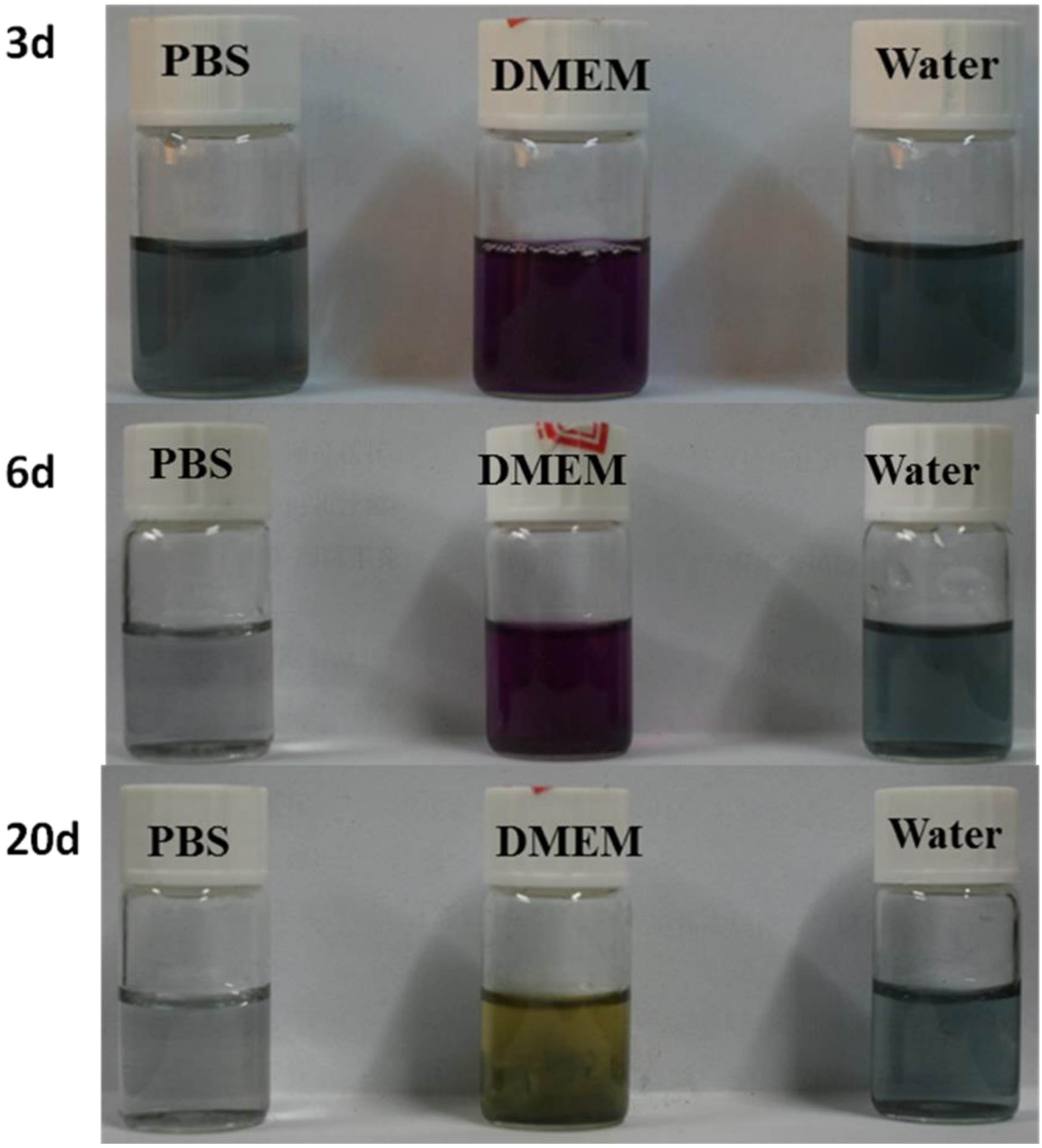

Fig. S4 CB[7]-GNSs is highly water-dispersible for over 3 days without visible aggregation and precipitation. CB[7]-GNSs is more stable in cell culture medium (DMEM) than in phosphate buffer solution (PBS, pH 7.4), and could be stored for more days in water than in both of DMEM and PBS.

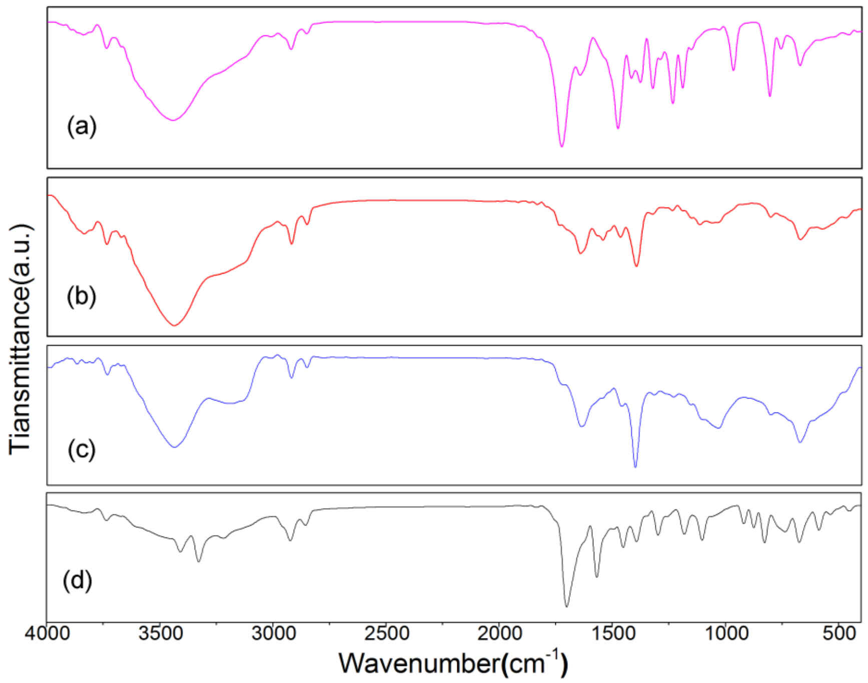

Fig. S5 FT-IR spectra of (a) free CB[7],(b) CB[7]-GNSs, (c) CB[7]-GNSs-AC, and (d) 6-Aminocoumarin (AC). FT-IR spectra were collected on Bruker Fourier spectrophotometer using KBr pellets.

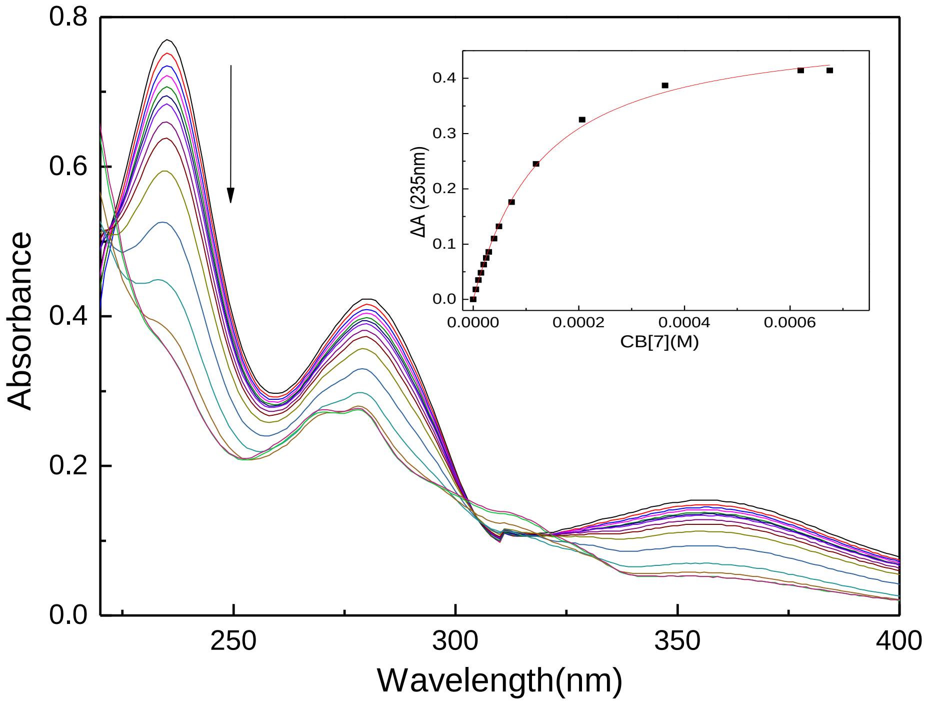

Fig. S6 UV-vis spectra from the titration of 6-Amioncoumarin (6-AC, 25 μM) with CB[7] (0-1.0 mM) in the aqueous CH_3CO_2Na buffer (pH = 4.74, 30 °C). Insert: plot of the absorbance at 235 nm for 6-AC as a function of CB[7] concentration, and the solid line represents the best least-squares fit of the data to a 1:1 binding model (K_a = 8.96 × 10^3 M^{-1}). All UV-vis spectra were collected on a UV-3600 Spectroscopy (Shimadzu, Japan).

Table S2 Association constants K_a of CB[7] and 6-AC at different temperatures.

T (˚C)	K_a (M^{-1}) (λ = 235nm)
20.0	1.36×10^4
25.0	1.08×10^4
30.0	8.96×10^3
35.0	7.20×10^3
40.0	6.00×10^3
45.0	4.59×10^3
50.0	3.30×10^3
55.0	2.00×10^3

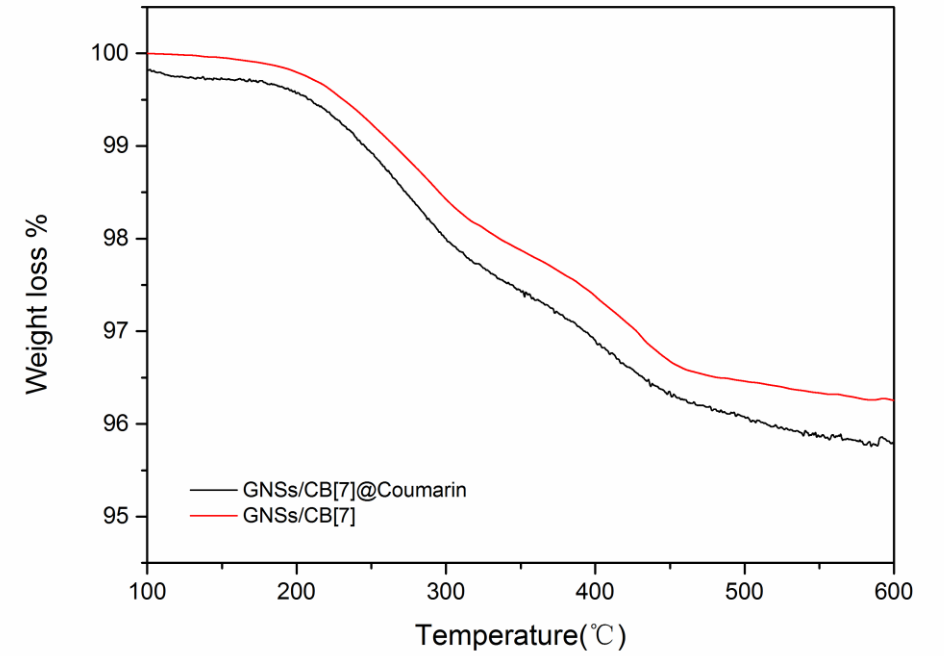

Fig. S7 TGA curves of CB[7]-GNSs with and without coumarin. TGA was taken with TA Q500 operated at 10 K/Min at nitrogen atmosphere.
